# Glutamine metabolism regulates FLIP expression and sensitivity to TRAIL in triple-negative breast cancer cells

**DOI:** 10.1038/s41419-018-0263-0

**Published:** 2018-02-12

**Authors:** Marta Mauro-Lizcano, Abelardo López-Rivas

**Affiliations:** 1 Centro Andaluz de Biología Molecular y Medicina Regenerativa-CABIMER, CSIC-Universidad de Sevilla-Universidad Pablo de Olavide, Avda Américo Vespucio 24, Sevilla, 41092 Spain; 2 Centro de Investigación Biomédica en Red-Oncología (CIBERONC), Carlos III Health Institute, Madrid, Spain

## Abstract

Glutamine plays an important role in the metabolism of tumor cells through its contribution to redox homeostasis, bioenergetics, synthesis of macromolecules, and signaling. Triple-negative breast cancers (TNBC) are highly metastatic and associated with poor prognosis. TNBC cells show a marked dependence on extracellular glutamine for growth. Herein we demonstrate that TNBC cells are markedly sensitized to tumor necrosis factor-related apoptosis-inducing ligand (TRAIL)-induced apoptosis upon glutamine deprivation. Upregulation of pro-apoptotic TRAIL receptor 2 (TRAIL-R2/DR5) and downregulation of FLICE-inhibitory protein (FLIP) are observed in glutamine-deprived TNBC cells. Activation of the amino-acid-sensing kinase general control nonderepressible 2 (GCN2) upon glutamine deprivation is responsible for TRAIL-R2 upregulation through a signaling pathway involving ATF4 and CHOP transcription factors. In contrast, FLIP downregulation in glutamine-deprived TNBC occurs by a GCN2-independent mechanism. Importantly, silencing FLIP expression by RNA interference results in a marked sensitization of TNBC cells to TRAIL-induced apoptosis. In addition, pharmacological or genetic inhibition of transaminases increases TRAIL-R2 expression and downregulates FLIP levels, sensitizing TNBC cells to TRAIL. Interestingly, treatment with l-asparaginase markedly sensitizes TNBC cells to TRAIL through its glutaminase activity. Overall, our findings suggest that targeting the glutamine addiction phenotype of TNBC can be regarded as a potential antitumoral target in combination with agonists of proapoptotic TRAIL receptors.

## Introduction

Oncogenic transformation leads to alterations in glutamine metabolism^1,2^ and makes transformed cells highly dependent on glutamine^3^. Triple-negative breast cancer (TNBC) is a heterogeneous group of breast cancer characterized by the absence of expression of estrogen (ER) and progesterone (PR) receptors, and lack of HER2 receptor gene amplification^4^. Patients with TNBC have a poor prognosis and a high rate of early relapse. TNBC still pose a major challenge in cancer management, being conventional chemotherapy the only therapeutic option^5^. Interestingly, different studies have demonstrated that TNBC cells are dependent on exogenous glutamine for survival and growth^6,7^. In this regard, inhibitors of glutamine transport and metabolism have been proposed as potential antitumor therapies^6,8^. However, targeting glutamine metabolism for cancer therapy may require identification of synergistic combinations with other therapeutic treatments to selectively target tumor cells in cancer patients and thus prevent unacceptable toxicity^9^.

Tumor necrosis factor-related apoptosis-inducing ligand (TRAIL) is a member of the TNF family that induces apoptosis selectively in a wide variety of cancer cells^10,11^. Binding of TRAIL to its pro-apoptotic receptors leads to the formation of a death-inducing signaling complex (DISC), where activation of initiator caspase-8 takes place^12^. At the DISC level, the apoptotic signal may be inhibited by cellular FLICE-inhibitory proteins FLIP_L_ and FLIP_S_^13^, which are short-lived inhibitory proteins^14^ expressed at high levels in breast cancers^15^. Interestingly, downregulation of FLIP levels is a common feature of various treatments that have been shown to sensitize different tumor cells to TRAIL-induced apoptosis^16–18^. The ability of TRAIL to induce apoptosis in tumor cells has prompted researches to further investigate its potential as an antitumor agent^19^. Nevertheless, many primary tumors are resistant to TRAIL and some tumors can acquire resistance during therapy^20^. In these cases, the use of TRAIL in combination with other treatments can result in additive or synergistic antitumor effects^21^.

In this study, we have investigated the regulation of TRAIL sensitivity by glutamine metabolism in TNBC cells. We report that inhibition of glutamine metabolism either by reducing extracellular glutamine concentration or by targeting glutamate-dependent transaminases synergizes with TRAIL in the activation of apoptosis in TNBC cells. Mechanistically, we demonstrate that glutamine consumption and catabolism are responsible for maintaining TRAIL-R2 and FLIP proteins at levels that prevent activation of the apoptotic machinery by TRAIL in TNBC cells. We propose that a combined strategy of targeting glutamine addiction and at the same time selectively activating the apoptotic machinery through the activation of proapoptotic TRAIL receptors would be a more efficient way of killing TNBC cells than either treatment alone.

## Results

### Glutamine deprivation markedly sensitizes triple-negative breast tumor cells to TRAIL-induced caspase-8 activation and apoptosis

**Fig. 1:**
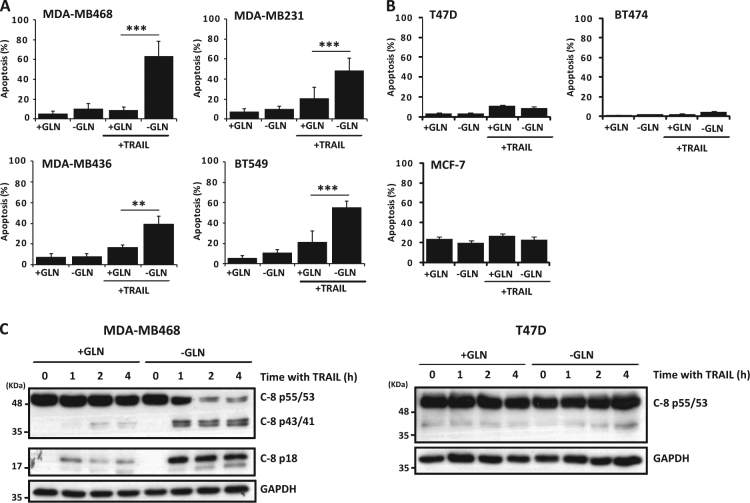
Glutamine deprivation sensitizes triple-negative breast tumor cells to TRAIL-induced caspase-8 activation and apoptosis. (**A**) TNBC and (**B**) non-TNBC cells were incubated for 24 h in medium with or without glutamine (2 mM) before incubation in the presence or absence of TRAIL for 24 h (100 ng/ml MDA-MB468, 50 ng/ml MDA-MB231, 10 ng/ml MDA-MB436, 100 ng/ml BT549, 500 ng/ml non-TNBC). Apoptosis was assessed as described in Material and Methods section. Error bars represent s.d. from three independent experiments. **P < 0.01, ***P < 0.001. (**C**) Cells were incubated in medium with or without glutamine as described in (**A**) before treating them with TRAIL for the times indicated. Caspase-8 activation was examined by western blotting. GAPDH was used as a protein loading control. Results shown are representative of at least three different experiments.

Cancer cells undergo reprogramming of glutamine metabolism to support redox homeostasis, bioenergetics, and biosynthesis of macromolecules, rendering cancer cells addicted to this non-essential amino-acid^22^. In this work, we have analyzed the regulation of sensitivity to TRAIL in cultures of TNBC and non-TNBC cells deprived of glutamine. Interestingly, when cultured in glutamine-free medium TNBC cell lines were sensitized to TRAIL-induced apoptosis (Fig. 1A). In sharp contrast, non-TNBC cell lines were markedly refractory to sensitization to TRAIL by glutamine deprivation (Fig. 1B).

To characterize the mechanism underlying glutamine deprivation-mediated sensitization to TRAIL-induced apoptosis in TNBC cells, we first examined activation of caspase-8, a key biochemical event that is triggered upon binding of TRAIL to its proapoptotic receptors at the cell surface^23^. Thus, we determined the processing of procaspase-8 into its 43/41 kDa intermediate proteolytic fragments and the generation of the mature p18 caspase-8 subunit in cells treated with TRAIL. Accordingly, the activation of caspase-8 induced by TRAIL was clearly enhanced in glutamine-deprived MDA-MB468 cells as early as one hour after TRAIL addition to the culture (Fig. 1C, left panel). In contrast, non-TNBC T47D cells were completely unresponsive to glutamine deprivation-induced sensitization to TRAIL-activated procaspase-8 processing (Fig. 1C, right panel). These results further confirmed the data on the different apoptotic response of TNBC and non-TNBC cells to TRAIL following glutamine deprivation.

### GCN2-mediated upregulation of TRAIL-R2/DR5 expression is dispensable for sensitization to TRAIL of glutamine-starved TNBC cells

**Fig. 2:**
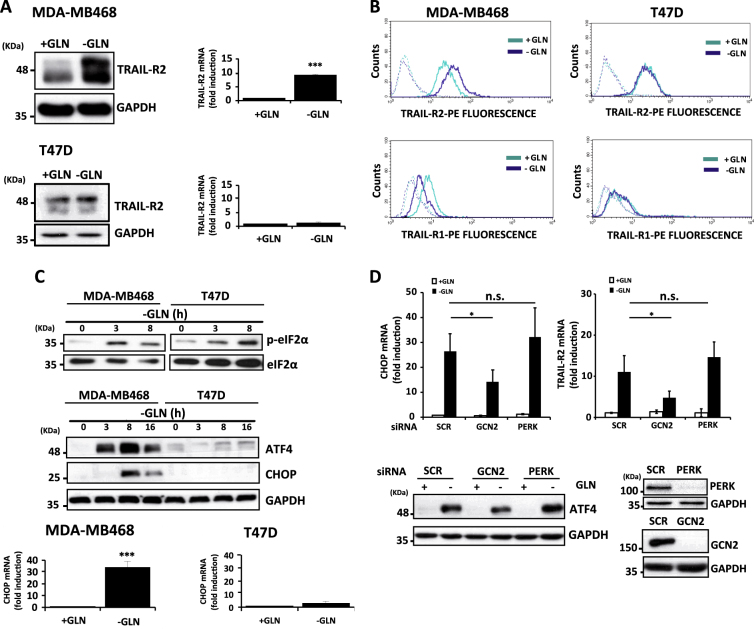
Glutamine deprivation up-regulates TRAIL-R2/DR5 expression in TNBC cells through the GCN2-activated pathway. MDA-MB468 and T47D cells were incubated with or without glutamine for 24 h. Following these treatments, (**A**) TRAIL-R2 levels were determined by western blotting and RT-qPCR, and (**B**) TRAIL-R1 and TRAIL-R2 cell surface levels were determined by flow-cytometry as described in the Materials and Methods section. Cells incubated with control IgG-PE antibody were used as a control for background fluorescence. Data are representative of at least three independent experiments. (**C**) Cells were incubated in the presence or absence of glutamine for the times indicated. Levels of the indicated proteins were assessed by western blotting. Results shown are representative of three independent experiments. CHOP levels were also determined by RT-qPCR following incubation of cells for 16 h in medium with or without glutamine. (**D**) MDA-MB468 cells were transfected either with a Scrambled oligonucleotide (SCR) or with siRNA targeting GCN2 or PERK for 30 h and then incubated with or without glutamine for 24 h. TRAIL-R2 and CHOP mRNA levels were determined by RT-qPCR and ATF4, GCN2 and PERK levels were assessed by western blotting. GAPDH was used as a protein loading control. In panels **A**, **C** and **D** error bars represent s.d. from three independent experiments. * *P* < 0.05, ***P < 0.001, n.s. not statistically significant.

To elucidate the mechanism underlying the sensitization of TNBC cells to TRAIL-induced apoptosis following glutamine deprivation, we initially determined the expression of proapoptotic TRAIL receptors in TNBC and non-TNBC cells after glutamine starvation. Incubation of MDA-MB468 cells in glutamine-free medium for 24 h induced the upregulation of TRAIL-R2/DR5 protein (Fig. 2A, left panel) and mRNA (Fig. 2A, right panel) expression. Importantly, cell surface levels of TRAIL-R2 were also upregulated upon glutamine deprivation in TNBC cells (Fig. 2B). On the contrary, surface levels of TRAIL-R1/DR4 were significantly reduced in TNBC cells deprived of glutamine (Fig. 2B). Interestingly, upregulation of TRAIL-R2/DR5 was not observed in T47D cells cultured in glutamine-free medium (Fig. 2A, B), which was in accordance with their refractoriness to undergo sensitization to TRAIL upon glutamine starvation.

**Fig. 3:**
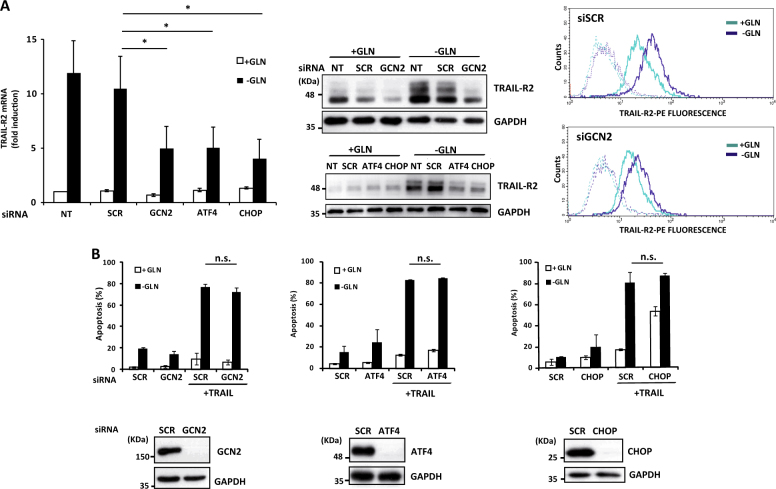
TRAIL-R2/DR5 upregulation is dispensable for sensitization to TRAIL upon glutamine deprivation. (**A**) MDA-MB468 cells were either non-transfected (NT) or transfected with siRNA of GCN2, ATF4, CHOP or Scrambled oligonucleotide (SCR) for 30 h and then incubated with or without glutamine for 24 h. TRAIL-R2 levels determined by RT-qPCR, western blotting and flow-cytometry as described in the Materials and Methods section. (**B**) MDA-MB468 cells were transfected with siRNA of GCN2, ATF4, CHOP or Scrambled (SCR) for 30 h and then incubated with or without glutamine for 24 h prior to treatment for 24 h in the presence or absence of TRAIL (100 ng/ml). Apoptosis was measured as described in Materials and Methods section. GCN2, ATF4 and CHOP levels were assessed by western blotting. GAPDH was used as a protein loading control. In **A** (left panel) and **B**, error bars represent s.d. from three independent experiments. *P < 0.05, n.s. not statistically significant

In response to glutamine deprivation cells can activate different adaptive responses to restore homeostasis^24^. The initial event in this signaling response is the phosphorylation of eukaryotic translation initiation factor 2 alpha (eIF2α) either by the general control nondepressible 2 (GCN2) kinase or the ER stress-activated kinase PERK to inhibit cap-dependent global protein synthesis while allowing the translation of specific mRNAs containing a short upstream open reading frame (uORF) in their 5′ untranslated region^25,26^. Among these mRNAs, translation of mRNA for activating transcription factor 4 (ATF4) is a key event in the adaptive response to stress^26^, that can also lead to cell death through the transcriptional activation of ATF4 target gene CHOP (GADD153)^27^, in turn a transcription factor for TRAIL-R2/DR5 gene expression^28^. To further characterize the mechanism underlying TRAIL-R2/DR5 upregulation in glutamine-deprived TNBC cells, we determined eIF2α phosphorylation and ATF4 expression in cells deprived of glutamine. Incubation of cells in glutamine-free medium induced clear increase in eIF2α phosphorylation in MDA-MB468 cells and less in T47D cells (Fig. 2C, upper panel). Accordingly, glutamine deprivation induced a marked upregulation of ATF4 protein expression that was followed by the induction of CHOP mRNA and protein (Fig. 2C, middle and lower panels) only in MDA-MB468 cells. In this respect, glutamine independence of T47D cells with a luminal phenotype has recently been correlated with elevated levels of glutamine synthetase (GLUL)^29^. Next, we investigated the role of the GCN2 and PERK pathways in TRAIL-R2/DR5 upregulation upon glutamine deprivation in MDA-MB468 cells. Whereas silencing PERK expression did not prevent ATF4, CHOP and TRAIL-R2/DR5 upregulation upon glutamine deprivation, GCN2 knockdown significantly inhibited this response in glutamine-starved cells (Figs. 2D and 3A right panel). Additional evidences for the role of the GCN2 pathway in TRAIL-R2/DR5 upregulation in glutamine-free medium were obtained in experiments using siRNAs to knockdown either ATF4 or CHOP expression (Fig. 3A, left and middle panels). To test the possibility that GCN2-mediated TRAIL-R2/DR5 upregulation was responsible for the sensitization of TNBC cells to TRAIL following glutamine deprivation, we determined the effect of GCN2, ATF4 or CHOP knockdown in apoptosis induced by TRAIL in glutamine-starved cells. Although starvation-mediated TRAIL-R2 upregulation was markedly reduced by silencing the expression of GCN2 pathway proteins (Fig. 3A), sensitization to TRAIL-induced apoptosis was not inhibited (Fig. 3B). Together, our results suggest that upregulation of TRAIL-R2 levels at the cell surface upon glutamine deprivation is dispensable for the observed sensitization to TRAIL in TNBC cells.

### Modulation of c-FLIP levels by glutamine regulates TRAIL sensitivity in TNBC cells

**Fig. 4:**
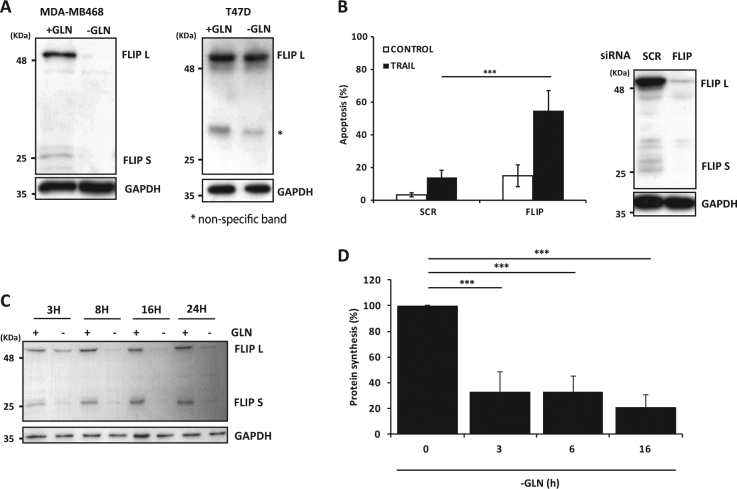
c-FLIP levels regulates TRAIL sensitivity in TNBC cells. (**A**) MDA-MB468 and T47D were incubated with or without glutamine for 24 h and FLIP levels were determined by western blotting. (**B**) MDA-MB468 cells were transfected with siRNA of FLIP or Scrambled (SCR) oligonucleotide for 30 h and then treated with or without TRAIL (100 ng/ml) for 24 h. Apoptosis was measured as described in Materials and Methods section. FLIP levels were determined by western blotting. Error bars represent s.d. from three independent experiments. ***P < 0.001. (**C**) MDA-MB468 cells were incubated in the presence or absence of glutamine for the indicated times and FLIP levels were determined by western blotting. GAPDH was used as a protein loading control (**D**) Protein synthesis was measured in MDA-MB468 cells after glutamine deprivation for the indicated times, as described in the Materials and Methods section. Error bars represent SD from three independent experiments. ***P < 0.001

The antiapoptotic proteins FLIP_L_ and FLIP_S_ are key regulators of TRAIL signaling^13^. To investigate whether these short-lived proteins could be involved in the sensitization process induced by glutamine deprivation in TNBC cells, we determined FLIP proteins levels in MDA-MB468 cells cultured in glutamine-free medium. Interestingly, glutamine deprivation induced a marked decrease in FLIP proteins levels in MDA-MB468 cells (Fig. 4A). In contrast, glutamine deprivation did not inhibit FLIP expression in cells of the non-TNBC cell line T47D (Fig. 4A). Collectively, these results closely correlated with sensitization to TRAIL in glutamine-free medium (Fig. 1A).

To demonstrate the importance of the loss of FLIP proteins caused by glutamine deprivation in the sensitization to TRAIL-induced apoptosis, we depleted both FLIP isoforms in MDA-MB468 cells with a siRNA oligonucleotide prior to treatment with TRAIL. As shown in Fig. 4B, silencing the expression of the two FLIP isoforms markedly sensitized MDA-MB468 cells to TRAIL-induced apoptosis. To further establish the role of FLIP downregulation after glutamine removal in the sensitization of TNBC cells to TRAIL, we generated MDA-MB231 cells that overexpress FLIP_L_ to maintain FLIP levels in cells deprived of glutamine. Results shown in Figure S1A demonstrate that sensitization to TRAIL-induced apoptosis was clearly inhibited in TNBC cells ectopically expressing FLIP_L_. Together, these results indicate that downregulation of cellular FLIP levels upon glutamine deprivation is a key step in the observed sensitization of TNBC cells to TRAIL-induced apoptosis.

In MDA-MB468 cells, FLIP downregulation was observed as early as 3 h after glutamine deprivation and downregulation of both FLIP isoforms was almost complete after 16 h of treatment (Fig. 4C). To examine whether modulation of FLIP expression by glutamine deprivation occurred at the mRNA level, we performed RT-qPCR analysis of FLIP mRNAs in glutamine-deprived cells. In contrast to the observed downregulation of FLIP proteins levels, incubation of cells in glutamine-free medium did not reduce the levels of FLIP mRNAs, which are indeed increased under these conditions (Fig. S1B). As both FLIP isoforms are short-lived proteins subject to ubiquitination and degradation by the proteasome in cells treated with antitumor drugs^14,16–18^, we determined whether glutamine deprivation might decrease their half-lives in MDA-MB468 cells. However, results shown in Figure S1C demonstrate that stabilities of both FLIP isoforms were not affected by removal of glutamine from the culture medium. Since neither FLIP mRNAs levels nor proteins half-lives were reduced in MDA-MB468 cells deprived of glutamine we next determined whether general protein synthesis was inhibited upon glutamine deprivation. Data shown in Fig. 4D demonstrate that protein synthesis was markedly inhibited as early as 3 h after glutamine removal from the medium, which closely correlated with FLIP downregulation (Fig. 4C).

The GCN2 signaling pathway is activated in response to amino acids starvation through the binding of uncharged transfer RNAs (tRNAs) to GCN2. As with other eIF2α kinases, GCN2 activation leads to severe inhibition of de novo protein synthesis while allowing translation of stress-regulated mRNAs to restore homeostasis^25^. To further characterize the mechanism underlying FLIP loss upon glutamine deprivation we next assessed the role of the GCN2 pathway in FLIP downregulation induced by glutamine deprivation in MDA-MB468 cells. However, loss of FLIP proteins in cells deprived of glutamine was not prevented by silencing the expression of GCN2, ATF4, or CHOP with siRNAs (Fig. S2A). These results further indicated that activation of the eIF2α kinase GCN2, a key effector of the integrated stress response to amino-acid deprivation, was not involved in the sensitization of TNBC cells to TRAIL following glutamine deprivation.

We also examined the role of the mechanistic target of rapamycin complex 1 (mTORC1), another important sensor of glutamine availability in mammalian cells^30^, in the regulation of FLIP levels and TRAIL sensitivity in TNBC cells. As shown in figure S2B, FLIP downregulation upon glutamine deprivation was accompanied by mTORC1 inhibition as determined by measuring phosphorylation of mTORC1 substrates p70S6K and 4EBP1. However, treatment of TNBC cells with mTORC1/mTORC2 inhibitor Torin-1 neither reduced FLIP levels (Fig. S2B) nor sensitized these cells to TRAIL-induced apoptosis (Fig. S2C). Likewise, mTORC1 inhibition by rapamycin did not sensitize TNBC cells to TRAIL (Fig. S2C). Collectively, these data indicated that mTORC1 inhibition was not responsible for the observed sensitization to TRAIL in glutamine deprived TNBC cells.

### Phamacological and genetic inhibition of transaminases downregulates FLIP expression and sensitizes TNBC cells to TRAIL

**Fig. 5:**
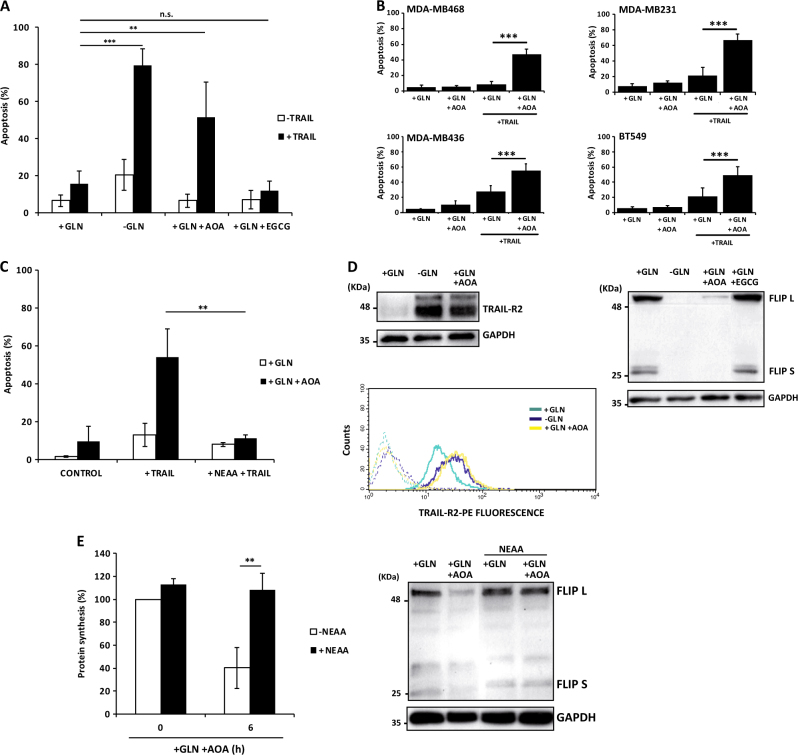
Pharmacological inhibition of transaminases downregulates FLIP expression and sensitizes TNBC cells to TRAIL. (**A**) MDA-MB468 cells were incubated for 24 h in medium with or without glutamine and the indicated additions prior to treatment in the presence or absence of TRAIL (100 ng/ml) for 24 h. Apoptosis was measured as described in Materials and Methods section. (**B**) TNBC cells incubated in glutamine-containing medium with or without AOA for 24 h were treated in the presence or absence of TRAIL for 24 h (100 ng/ml MDA-MB468, 50 ng/ml MDA-MB231, 10 ng/ml MDA-MB436, 100 ng/ml BT549) and apoptosis was then determined. (**C**) Apoptosis in MDA-MB468 cells incubated in medium with glutamine and AOA in the presence or absence of non-essential amino acids (NEAA) before treatment with or without TRAIL (100 ng/ml). (**D**) MDA-MB468 cells were treated as indicated and TRAIL-R2 levels were assessed by western blotting or flow-cytometry. FLIP levels were determined by western blotting. Results shown are representative of three independent experiments. (**E**) Protein synthesis in MDA-MB468 cells incubated in glutamine-containing medium and the metabolic inhibitor AOA in presence or absence of NEAA for 6 h (left panel). (Right panel) MDA-MB468 cells were incubated as indicated for 24 h and FLIP levels were assessed by western blotting. GAPDH was used as a protein loading control. In panels **A**,** B**, **C** and** E**, error bars represent s.d. from three independent experiments. **P < 0.01, ***P < 0.001, n.s. not statistically significant.

Interestingly, addition of α-ketoglutarate (α-KG), a key metabolite of glutamine metabolism, to glutamine-deprived MDA-MB468 cells fully prevented FLIP downregulation and sensitization to TRAIL-induced apoptosis (Fig. S3A). In the cells, reductive amination of α-KG produces glutamate, a precursor in the biosynthesis of non-essential amino acids (NEAA), required for protein synthesis, through the activity of transaminases^31^. Together, these data suggested that targeting transaminases with specific inhibitors may sensitize TNBC cells to TRAIL. In line with this, we observed that treatment of MDA-MB468 cells with aminooxyacetate (AOA), a general inhibitor of transaminases^32^, effectively sensitized these cells to TRAIL in glutamine-containing medium (Fig. 5A). In contrast, epigallocatechin gallate (EGCG) a potent inhibitor of glutamate dehydrogenase did not show any sensitizing effect in TRAIL-induced apoptosis (Fig. 5A). Moreover, as shown in Fig. 5B, sensitization to TRAIL by AOA was also observed in other TNBC cell lines. Interestingly, AOA-induced sensitization to TRAIL was abrogated by adding NEAA to the culture medium (Fig. 5C), further supporting a role of NEAA biosynthesis in the regulation of sensitivity to TRAIL in TNBC cells. We next investigated the mechanism underlying AOA-mediated sensitization to TRAIL-induced apoptosis by determining the expression levels of TRAIL-R2 and FLIP in cells treated with the transaminases inhibitor in glutamine-containing medium. As shown in Fig. 5D, treatment of MDA-MB468 cells for 24 h with AOA significantly increased both total and cell surface TRAIL-R2 levels. These results suggested that inhibition of transaminases-mediated NEAA biosynthesis in TNBC cells may activate a stress response leading to TRAIL-R2 upregulation. Results shown in Figures S3B and S3C confirmed this hypothesis since addition of NEAA to the culture medium completely inhibited AOA-induced ATF4, CHOP, and TRAIL-R2 upregulation. Remarkably, treatment with AOA, but not with EGCG, induced a marked loss of FLIP expression in MDA-MB468 cells (Fig. 5D). Importantly, NEAA supplementation fully prevented AOA-induced inhibition of protein synthesis and FLIP downregulation (Fig. 5E), further supporting a role of NEAA biosynthesis through transaminases in the control of FLIP levels in TNBC cells.

**Fig. 6:**
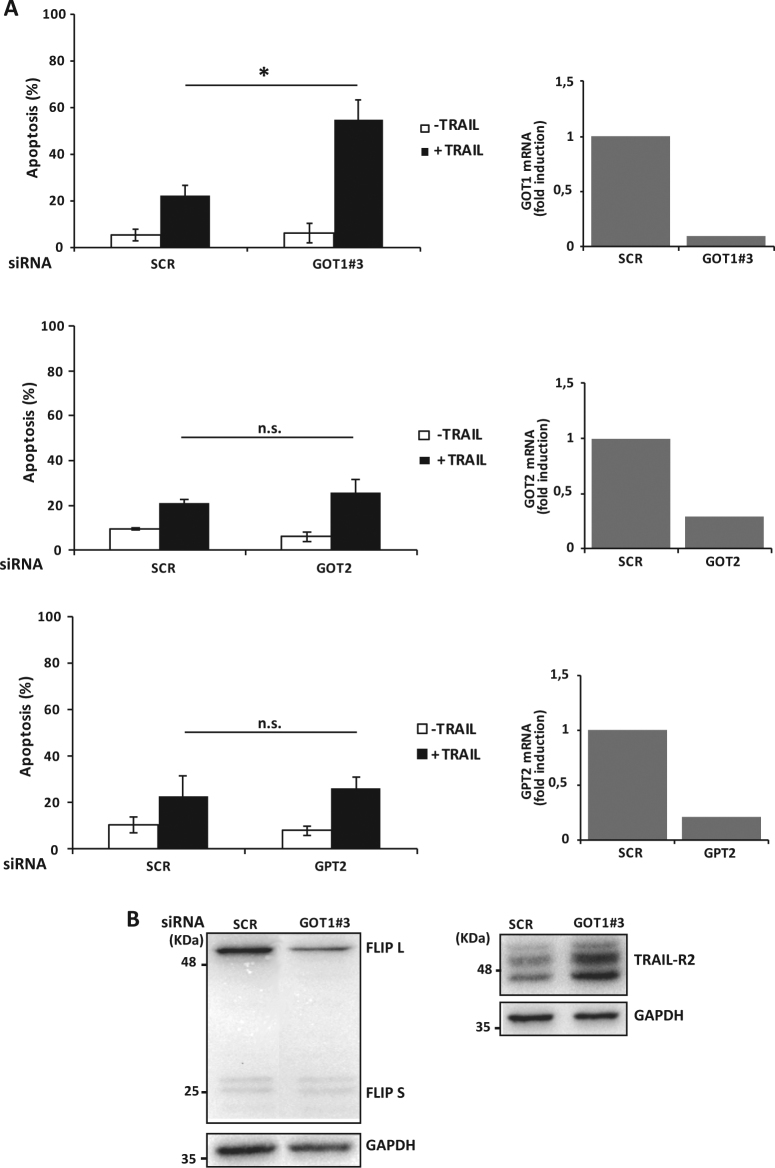
Genetic inhibition of GOT1 downregulates FLIP expression and sensitizes TNBC cells to TRAIL. (**A**) MDA-MB468 cells were transfected either with a Scrambled oligonucleotide (SCR) or siRNAs of GOT1, GOT2 or GPT2 for 30 h and then treated with or without TRAIL (100 ng/ml). GOT1, GOT2 and GPT2 mRNA levels were determined by RT-qPCR. Apoptosis was measured as described in Materials and Methods section. Error bars represent s.d. from three independent experiments. *P < 0.05. n.s. not statistically significant. (**B**) MDA-MB468 cells were transfected either with a Scrambled oligonucleotide (SCR) or siRNA of GOT1 for 30 h. FLIP and TRAIL-R2 levels were assessed by western blotting. GAPDH was used as a protein loading control. Data shown are representative of three independent experiments

To confirm the involvement of transaminases in the regulation of TRAIL sensitivity of TNBC cells, we performed genetic inhibition of different transaminases using siRNAs that efficiently silenced their expression in MDA-MB468 cells (Fig. 6A and Supplementary Figure S3C). We next examined the sensitivity of these cells to TRAIL-induced apoptosis following individual knockdown of the various transaminases. The cytoplasmic form of glutamate-oxoloacetate transaminase (GOT1) transfers nitrogen from glutamate to oxoloacetate to produce aspartate and α-KG. Interestingly, results shown in Fig. 6A and Supplementary Figure S3C demonstrate that silencing the expression of GOT1 with different siRNAs significantly sensitized MDA-MB468 cells to TRAIL. In contrast, knockdown of the mitochondrial forms of either glutamate-oxoloacetate transaminase (GOT2) or glutamate-pyruvate transaminase (GPT2) did not sensitize these cells to TRAIL (Fig. 6A). Furthermore, results shown in Fig. 6B indicate that the sensitizing effect of GOT1 knockdown in TRAIL-induced apoptosis was preceded by FLIP downregulation and TRAIL-R2 upregulation, further supporting a role of cytosolic GOT1 in restraining TRAIL-induced apoptosis in TNBC cells.

### l-Asparaginase treatment sensitizes TNBC cells to TRAIL-induced apoptosis

**Fig. 7:**
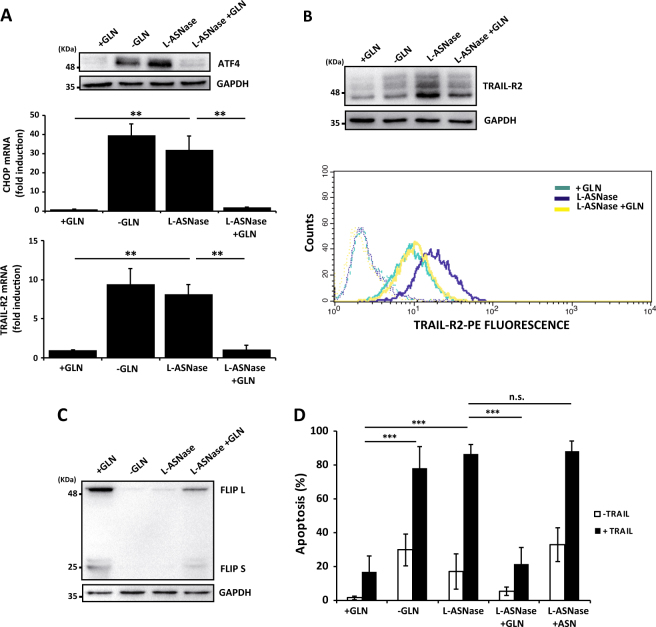
Treatment with l-Asparaginase sensitizes TNBC cells to TRAIL. MDA-MB468 cells were incubated in medium with glutamine (+GLN), without glutamine (-GLN) or with glutamine and l-Asparaginase (l-ASNase) for 48 h. Glutamine (2 mM) was added to some cultures for the last 6 h of incubation with l-ASNase (l-ASNase + GLN). (**A**) ATF4 levels were assessed by western blotting. CHOP and TRAIL-R2 mRNA levels were determined by RT-qPCR. Error bars represent SD from three independent experiments. **P < 0.01. (**B**) TRAIL-R2 levels were assessed either by western blotting or by flow-cytometry with a TRAIL-R2-PE antibody as described in the Materials and Methods section. Cells incubated with control IgG-PE antibody were used as a control for background fluorescence. (**C**) FLIP levels were assessed by western blotting. GAPDH was used as a protein loading control. Data are representative of at least three independent experiments (panels **B** and **C**). In (**D**) MDA-MB468 cells incubated as described above were treated for 24 h in the presence or absence of TRAIL (100 ng/ml) and apoptosis was measured as described in Materials and Methods section. Error bars represent s.d. from three independent experiments. ***P < 0.001. n.s. not statistically significant.

Although early clinical trials of glutamine metabolism inhibitors have revealed systemic toxicity, targeting glutamine dependency of tumor cells remains a promising approach to treat cancer patients^24^. It has been suggested that lowering blood glutamine levels with l-Asparaginase (l-ASNase) is in part responsible for its anti-leukemic action^33^. We first determined the effect of l-ASNase treatment on the activation of the ATF4/CHOP/TRAIL-R2 stress response in TNBC cells. Incubation of MDA-MB468 cells in glutamine-containing culture medium in the presence of *Escherichia coli* l-ASNase induced the expression of ATF4, CHOP and TRAIL-R2 (Fig. 7A, B). Interestingly, addition of 2 mM glutamine in the last 6 h of treatment with l-ASNase completely inhibited this stress response, which suggested that glutaminase activity of l-ASNase was responsible for the activation of the ATF4/CHOP/TRAIL-R2 pathway. Remarkably, l-ASNase treatment downregulated FLIP expression levels (Fig. 7C) and late addition of glutamine to l-ASNase-treated cell cultures markedly inhibited FLIP downregulation (Fig. 7C). These results prompted us to investigate whether l-ASNase treatment would sensitize TNBC cells to TRAIL-induced apoptosis. Incubation of MDA-MB468 cells with l-ASNase in glutamine-containing medium strongly sensitized these cells to TRAIL-induced apoptosis (Fig. 7D). Importantly, addition of glutamine 6 h before TRAIL treatment completely abrogated sensitization to TRAIL (Fig. 7D). In contrast, asparagine (ASN) supplementation for 6 h prior to TRAIL addition did not inhibit TRAIL-induced apoptosis (Fig. 7D). Collectively, our results clearly demonstrated that glutaminase activity of *E. coli* l-ASNase has a synergistic effect with TRAIL in the activation of an apoptotic cell death process in TNBC cells.

## Discussion

Clinical and molecular heterogeneity together with the lack of targeted therapies in patients with triple-negative breast cancer have prompted the search for new targets to treat these patients^5^. Recent evidences have indicated that the increased uptake and metabolism of glutamine in TNBC cells may provide these tumor cells with intermediates for nucleotide and protein synthesis, redox homeostasis and mitochondrial energy metabolism required for proliferation^6, 7,29^. In this respect, the dependence of TNBC cells on glutamine uptake and metabolism have made them attractive therapeutic targets^6,8^. However, initial clinical trials with agents targeting glutamine metabolism revealed unacceptable systemic toxicity, suggesting that lower doses of glutamine antimetabolites in combination with other targeted therapies may be required to selectively target TNBC.

Recent works have suggested a role of glutamine metabolism in the control of TRAIL-R2 expression and TRAIL sensitivity in different tumor cells^34–36^, although the underlying mechanism remained largely unknown. Our results clearly show that inhibiting glutamine utilization by TNBC cells with a basal phenotype markedly increases their sensitivity to TRAIL-induced apoptosis. In contrast, non-TNBC cell lines with a luminal phenotype were markedly refractory to sensitization to TRAIL upon inhibition of glutamine utilization. Differences in glutamine dependence have been observed between basal and luminal cell lines^6, 7,29^. Moreover, glutamine independence of breast tumor cells with a luminal phenotype was associated with elevated expression of glutamine synthetase (GLUL) and reduced glutaminase (GLS) levels^29^. Interestingly, differential expression of GLUL and GLS were also observed between luminal A and basal primary tumors^29^. We demonstrate for the first time that TRAIL-R2 and FLIP levels are critically regulated by glutamine metabolism in TNBC cells through different mechanisms. Whereas TRAIL-R2 upregulation upon glutamine deprivation depends on the activation of the GCN2-mediated signaling pathway, downregulation of FLIP levels results from the GCN2-independent inhibition of general protein synthesis, a likely consequence of the lack of non-essential proteinogenic amino acids in glutamine-starved cells^31^. Our data also demonstrate that inhibiting glutamate catabolism through non-essential amino-acid-generating transaminases, but not through glutamate dehydrogenase mimics the effects of glutamine deprivation on FLIP levels and sensitivity to TRAIL. Our data also show that cytosolic aspartate aminotransferase GOT1, an enzyme elevated in TNBC cell lines^37^, is involved in maintaining FLIP and TRAIL-R2 levels in TNBC cells in glutamine-containing medium. Together, these results further support a key role of transaminases in the control of the extrinsic pathway of apoptosis in TNBC cells. Interestingly, a recent report has demonstrated a strong correlation between transaminase pathway activity and proliferation in human breast tumors^38^. Thus, coupling glutamine anaplerosis to NEAA synthesis is an important step in the reprogramming of metabolism to sustain the biosynthetic demands of highly proliferative human breast tumors^38^ and transaminases have been proposed as potential targets for antitumor treatments in breast cancer^32,37^. However, metabolic redundancy between transaminases and glutamate dehydrogenases to produce the anaplerotic intermediate α-ketoglutarate, might lead to the development of tumor resistance to inhibitors of transaminases in monotherapy^38^. Importantly, our results suggest that reducing FLIP levels by transaminase inhibition and simultaneously activating proapoptotic TRAIL receptors might allow TNBC cells to reach the threshold for apoptosis induction, facilitating tumor suppression.

Although in vitro sensitivity to l-ASNase has been demonstrated in cells from different solid tumors, clinical use has been mainly restricted to pediatric and adult patients with acute lymphoblastic leukemia (ALL) and other lymphoid malignancies^39^. Interestingly, our results demonstrate that glutaminase activity of *E. coli* l-ASNase is responsible for the observed upregulation of TRAIL-R2, downregulation of FLIP and sensitization to TRAIL-induced cell death in TNBC treated with l-ASNase. Consistent with our in vitro findings in TNBC cells, clinical studies have indicated that glutaminase activity of l-ASNase correlates with the clinically beneficial effect in patients with ALL^39^. Although serious side-effects has been reported in long-term clinical treatments with l-ASNase^40^, the synergism with TRAIL demonstrated in our study may potentially reduce the duration of l-ASNase treatment and therefore minimize the risk of undesired effects in patients.

Despite possible liver toxicity of His-tagged TRAIL^41^, non-tagged versions of recombinant TRAIL and agonists antibodies against proapoptotic TRAIL receptors have been used in clinical trials, with broad tolerability^42^. Unfortunately, clinical benefit of these therapeutic approaches has so far been rather limited likely due, among other reasons, to low agonistic activity of these agents and resistance of most primary human tumors to them^21^. Thereafter, second-generation formulations of TRAIL have been generated to increase the agonistic bioactivity of this death ligand^43^. In this context, our data on the regulation of the TRAIL system by the glutamine metabolism in TNBC cells warrant further studies to ascertain the feasibility of inhibiting glutamine utilization for therapeutic intervention in TNBC, in combination with these novel agonists of proapoptotic TRAIL receptors.

## Materials and methods

### Reagents and Antibodies

Soluble human His-tagged recombinant TRAIL was generated in our laboratory as described^44^. Anti-caspase 8 antibody was generously provided by Dr. Gerald Cohen (Leicester University, UK). Antibodies against p-AKT^Ser473^, AKT, p-P70S6K, P70S6K, PERK (C33E10), eIF2α (D7D3), p-eIF2α and CHOP (D46F1) were obtained from Cell Signaling Technology (CA, USA). Antibodies against ATF4, GAPDH, and GCN2 were obtained from Santa Cruz Technology (Santa Cruz, CA, USA). Anti-TRAIL-R2 antibody was purchased from R&D Systems (Minneapolis, USA). Anti-FLIP (7F10) antibody was from Enzo Life Sciencies (Farmingdale, NY, USA). p4E-BP1 and 4E-BP1 antibodies were from Upstate Millipore (NY, USA). FITC-conjugated secondary antibodies were from DAKO (Cambridge, UK). Anti-TRAIL-R1-PE and anti-TRAIL-R2-PE monoclonal antibodies for surface receptor analysis were from Biolegend (San Diego, CA, USA). Aminooxyacetate, non-essential amino-acid solution (100×), cycloheximide, dimethyl 2-oxoglutarate (α-KG), and l-Asparaginase from E.Coli were from Sigma-Aldrich (St. Louis, MO, USA). Torin1 was purchased from TOCRIS Bioscience (Bristol, UK). Rapamycin was purchased from LC laboratories (Woburn, MA, USA). Epigallocatechin Gallate (EGCG) was purchased from Selleck Chemicals (Houston, Texas, USA).

### Cell culture

Breast tumor cell lines were maintained in DMEM (MDA-MB468 and MDA-MB436) or RPMI 1640 (MDA-MB231, MCF-7, BT474 and BT549) medium with 2 mM l-glutamine, penicillin (50 U/ml), streptomycin (50 μg/ml), and with 10% fetal bovine serum. T47D cell line was maintained in RPMI medium with 10% fetal bovine serum, 2 mM l-glutamine, 5 mM glucose, penicillin (50 U/ml), and streptomycin (50 μg/ml). Cells were maintained at 37 °C in a 5% CO_2_-humidified, 95% air incubator. BT-549, MDA-MB436, T47D, and BT474 cell lines were purchased from Cell Lines Service (CLS, Eppelheim, Germany). MDA-MB468, MDA-MB231, and MCF-7 cell lines were obtained from Dr. Joaquin Arribas (Vall D’Hebron Institute of Oncology, Barcelona, Spain). Glutamine deprivation experiments were performed on all cell lines in DMEM medium lacking NEAA, with 10% dialyzed fetal bovine serum, penicillin (50 U/ml), and streptomycin (50 μg/ml). When indicated, glutamine was added back at final concentration of 2 mM.

### Retroviral vectors and virus production

FLIP_L_ (in pCR3.V64 vector, a kind donation of Dr. J. Tschopp, University of Lausanne) was cloned into *Bam*HI/*Sal*I sites of pBabepuro. Retroviruses for protein overexpression were produced by transfection of HEK293-T cells by calcium phosphate method with the corresponding retroviral vectors. Retrovirus-containing supernatants were collected 48 h after transfection and concentrated by ultracentrifugation at 22,000 r.p.m. for 90 min at 4°C.

### Generation of MDA-MB231 cells over-expressing FLIP(L)

MDA-MB231 cells were plated at 3.5 × 10^5^ cells per 10 cm dish and infected with the retroviruses mentioned above. Stable populations of MDA-MB231 cells infected with retroviruses were obtained after selection in culture medium containing puromycin (1.5 µg/ml) during 48 h.

### RNA interference

siRNAs against ATF4, CHOP, FLIP, GCN2, GOT1, GOT2, GPT2, PERK, and non-targeting scrambled control oligonucleotide (Table), were synthesized by Sigma (St. Louis, MO). Cells were transfected with siRNAs using DharmaFECT-1 (Dharmacon) as described by the manufacturer. After 6 h, transfection medium was replaced with regular medium and cells were further incubated for 30 h before treatments.

**Table:** 

ATF4	5′-GCCUAGGUCUCUUAGAUGAdTdT-3′
CHOP pool	5′-AGGGAGAACCAGGAAACGGAA-3′
	5′-ACGGCUCAAGCAGGAAAUCGA-3′
	5-′AAGGAAGUGUAUCUUCAUACA-3′
	5′-CAGCUUGUAUAUAGAGAUUGU-3′
FLIP	5′-CUUUGGGUGAUCUACGUUAdTdT-3′
GCN2	5′-CAGCAGAAAUCAUGUACGAUU-3′
GOT1#1	5′-GGGCUUCCUGAAUGAUCUGdTdT-3′
GOT1#2	5´-GCUAAUGACAAUAGCCUAAAUdTdT-3´
GOT1#3	5´-GCGUUGGUACAAUGGAACAAAdTdT-3´
GOT2	5′-GGGACACCAAUAGCAAAAAdTdT-3′
GPT2	5′-CGAGUGUGGUUACAGAGGAdTdT-3′
PERK	5′-CAAACUGUAUAACGGUUUAdTdG-3′
Scrambled control	5′-GAGCGCUAGACAAUGAAG-3′

### Protein synthesis

Protein synthesis was measured by the incorporation of [^3^H]leucine (2μCi/ml) into acid-precipitable material. After pulse-labeling (2 h), the medium was aspirated, and the cells were washed three times with phosphate-buffered saline and treated with 1 ml of ice-cold 5% trichloroacetic acid. The cells were kept in the cold for 20 min, washed once with cold 5% trichloroacetic acid and twice with ethanol, and then dissolved in 0.1 M NaOH, 2% Na_2_CO_3_, 0.1% SDS for measurement of radioactivity in a Beckman scintillation counter.

### Real time-qPCR

mRNA expression was analyzed in triplicate by RT-qPCR on the ABI Prism7500 sequence detection system using predesigned assay-on-demand primers and probes (Applied Biosystems). Hypoxanthine-guanine phosphoribosyltransferase (HPRT1 Hs01003267_m1) was used as an internal control and mRNA expression levels were given as fraction of mRNA levels in control cells. Primers and probes used were: CHOP (Hs01090850_m1), FLIP(L) (AIN1EV0), FLIP(S) (Ss03391532_m1), TRAIL-R2 (Hs00366278_m1), GOT1 (Hs00157798_m1), GOT2 (Hs00905827_g1) and GPT2 (Hs00370287_m1).

### Determination of apoptosis

Cells (3 × 10^5^/well) were treated in six-well plates as indicated in the figure legends. After treatment, hypodiploid apoptotic cells were detected by flow cytometry according to published procedures^45^. Basically, cells were washed with cold phosphate-buffered saline (PBS), fixed in 70% cold ethanol and then stained with propidium iodide while treating with RNAse. Quantitative analysis of the cell cycle and sub-G1 cells was carried out in a FACSCalibur cytometer using the Cell Quest software (Becton Dickinson, Mountain View, CA, USA).

### Analysis of TRAIL receptors by flow cytometry

Cells were detached with trypsin solution and resuspended in growth media. After incubation for 15 min under cell culture conditions (37°C in a 5% CO_2_-humidified, 95% air incubator), cells were washed with ice-cold phosphate-buffered saline (PBS) and resuspended in PBS. Cells were then labeled either with 5 μg/ml of anti-TRAIL-R1-PE, anti-TRAIL-R2-PE or an IgG-PE control antibody for 30 min on ice and darkness. Quantitative analysis of the receptor cell surface expression was carried out in a FACSCalibur cytometer using the Cell Quest Software (Becton Dickinson, Mountain View, CA, USA).

### Immunoblot analysis of proteins

Cells (3 × 10^5^) were washed with PBS and lysed in TR3 lysis buffer (3% SDS, 10% Glycerol, 10 mM Na_2_HPO_4_). Then, lysates were sonicated and protein content was measured with the Bradford reagent (Bio-Rad Laboratories, USA), before adding Laemmli sample buffer. Proteins were resolved on SDS-polyacrylamide minigels and detected as described previously^45^. GAPDH was used as protein loading control.

### Statistical analysis

All data are presented as the mean ± s.d. of at least three independent experiments. The differences among different groups were determined by the Student’s *t*-test. *P* < 0.05 was considered significant.

## Electronic supplementary material

Supplementary material
