# Supplementary material for: Glutamine metabolism regulates FLIP expression and sensitivity to TRAIL in triple-negative breast cancer cells
Source: Cell Death Dis. 2018 Feb 12;9(2):205. doi: 10.1038/s41419-018-0263-0 (PMC5833863; doi:10.1038/s41419-018-0263-0)
Supplement: Supplementary file 1 — Supplementary material [file 41419_2018_263_MOESM1_ESM.pdf]

Supplementary figure legends

Figure S1. Control of FLIP levels and apoptosis upon glutamine deprivation in TNBC cells. (A) FLIP(L)-overexpressing MDA-MB231 cells were incubated with or without glutamine for 24 hours prior to treatment with TRAIL for 24 hours. FLIP(L) levels were measured by Western blotting. GAPDH was used as a protein loading control. Apoptosis was assessed as described in the Materials and Methods section. Error bars represent SD from three independent experiments. ** $P<0.01$. (B) MDA-MB468 cells were treated with or without glutamine for 24 hours. FLIP mRNA levels were determined by RT-qPCR. Error bars represent SD from three independent experiments. (C) MDA-MB468 cells were treated with or without glutamine for 3 hours. Cells were then treated with cycloheximide (CHX) for the indicated times. FLIP levels were determined by Western blotting and quantified with Image Quant 5.2 software. FLIP protein levels were normalized to the GAPDH loading control and referred to the untreated condition.

Figure S2. Role of GCN2 and mTOR in the control of FLIP levels and sensitivity to TRAIL-induced apoptosis upon glutamine deprivation. (A) MDA-MB468 cells were either non-transfected (NT) or transfected with siRNA of GCN2, ATF4, CHOP or Scrambled oligonucleotide (SCR) for 30 hours and then incubated with or without glutamine for 24 hours. FLIP levels were assessed by Western blotting. Results shown are representative of three independent experiments. (B) MDA-MB468 cells were incubated in medium with or without glutamine in presence or absence of Torin-1 for 24 hours. FLIP, p-p70S6K,

p70S6K, p-Akt, Akt, p-4EBP1 and 4EBP1 levels were determined by Western blotting. (C) MDA-MB468 cells were incubated in medium with glutamine (+GLN), without glutamine (-GLN), with glutamine and Torin-1 (+GLN +Torin-1) or with glutamine and Rapamycin (+GLN +RAPA) for 24 hours before treatment with or without TRAIL (100ng/ml) Apoptosis was determined as described in Materials and Methods. Error bars represent SD from three independent experiments. *** P<0.001. n.s. not statistically significant. p-p70S6K and p70S6K levels were determined by Western blotting. GAPDH was used as protein loading control.

Figure S3. Role of transaminases in the control of FLIP levels and apoptosis in TNBC cells. (A) MDA-MB468 cells were incubated for 24 hours in medium with or without glutamine in presence or absence of α-ketoglutarate (αKG) prior to treatment with TRAIL (100ng/ml). Apoptosis was determined as described in Materials and Methods. FLIP levels were assessed by Western blotting. (B and C) MDA-MB468 cells were treated for 24 hours with or without the transaminase inhibitor AOA in glutamine-containing medium, in the presence or absence of NEAA. In (B), TRAIL-R2 mRNA levels were determined by RT-qPCR. (C) CHOP mRNA levels were determined by RT-qPCR. ATF4 and CHOP protein levels were determined by Western blotting. GAPDH was used as a protein loading control. (D) MDA-MB468 cells were transfected with a Scrambled oligonucleotide (SCR) or siRNA of GOT1 for 30 hours and then treated with or without TRAIL (100ng/ml). Apoptosis was measured as described in Materials and Methods section. GOT1 mRNA levels were determined by RT-qPCR. In panels A, B, C and D error bars represent SD from

three independent experiments. * $P<0.05$, *** $P<0.001$. n.s. not statistically significant.

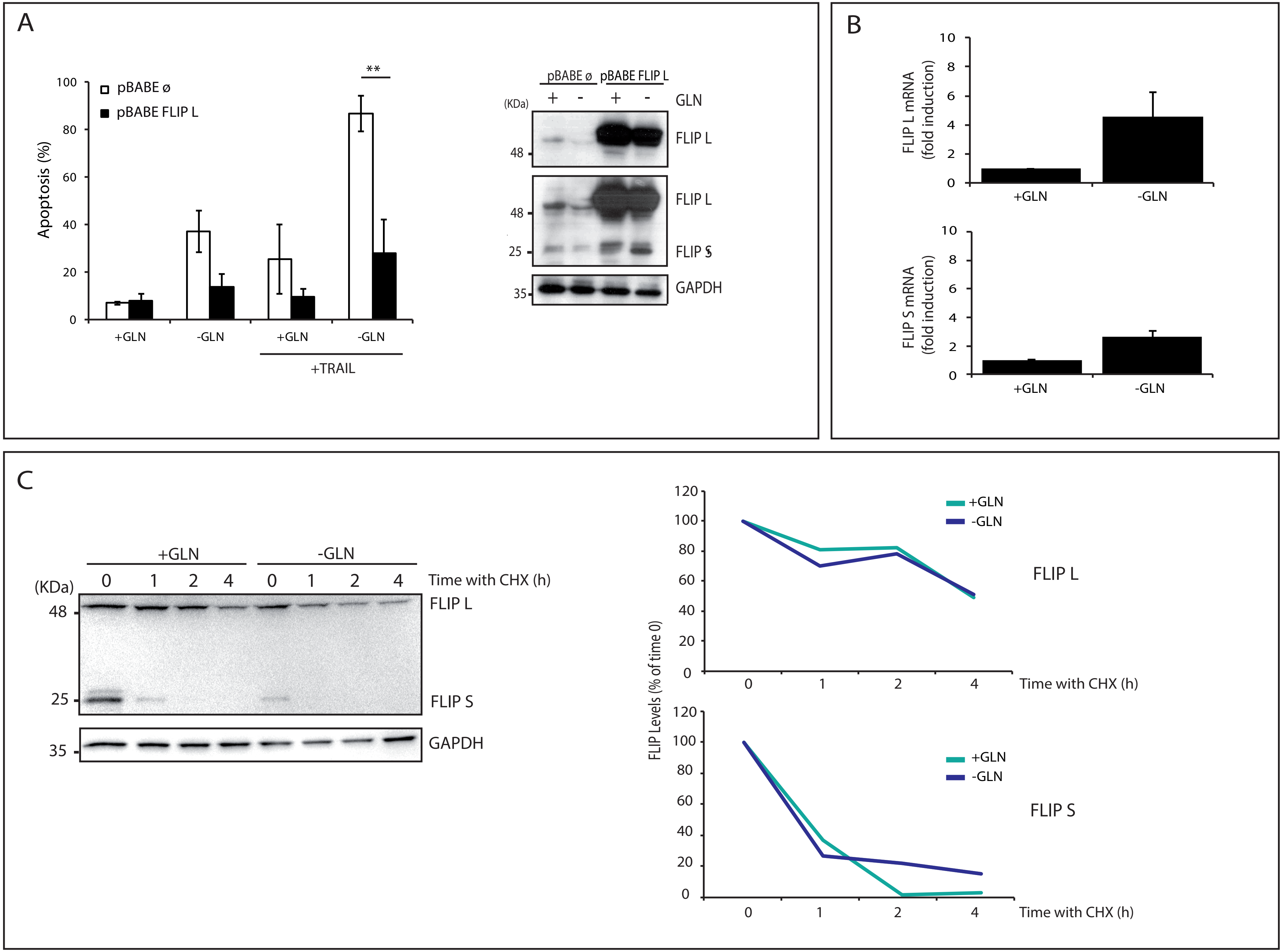
A
100
80
60
40
20
0
Apoptosis (%)
pBABE ø
pBABE FLIP L
**
+GLN
-GLN
+GLN
-GLN
+TRAIL
pBABE ø
pBABE FLIP L
(KDa)
GLN
48
FLIP L
48
25
FLIP L
FLIP S
35
GAPDH
B
FLIP L mRNA (fold induction)
10
8
6
4
2
0
+GLN
-GLN
FLIP S mRNA (fold induction)
+GLN
-GLN
C
+GLN
-GLN
(KDa)
0 1 2 4 0 1 2 4
Time with CHX (h)
48
FLIP L
25
FLIP S
35
GAPDH
FLIP Levels (% of time 0)
120
100
80
60
40
20
0
FLIP L
FLIP S
Time with CHX (h)

Figure S1

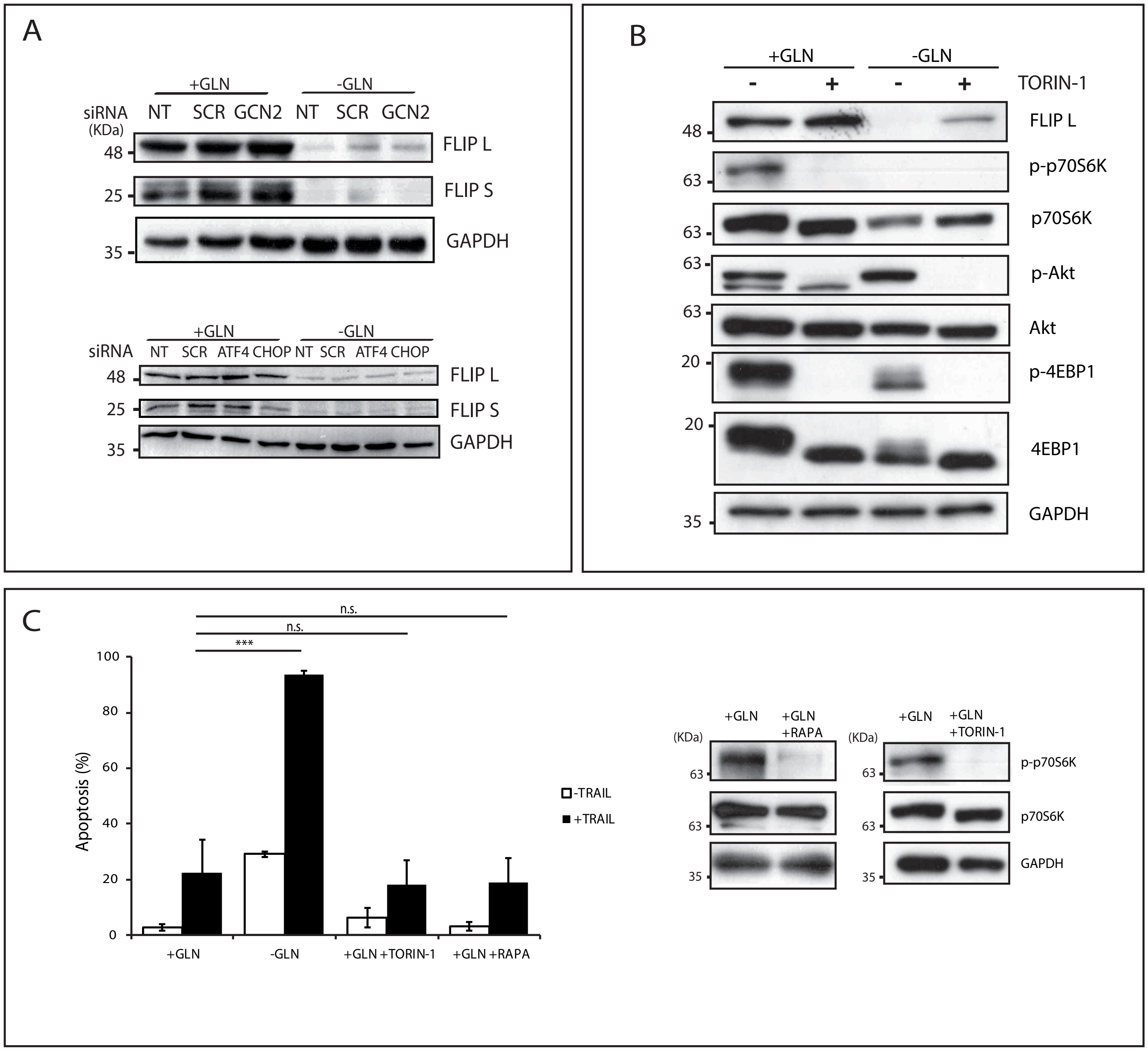
A
+GLN
-GLN
siRNA
NT
SCR
GCN2
(KDa)
48
25
35
FLIP L
FLIP S
GAPDH
ATF4
CHOP
B
TORIN-1
p-p70S6K
p70S6K
p-Akt
Akt
p-4EBP1
4EBP1
63
20
C
n.s.
***
Apoptosis (%)
-TRAIL
+TRAIL
+GLN +TORIN-1
+GLN +RAPA
+RAPA
+TORIN-1

Figure S2

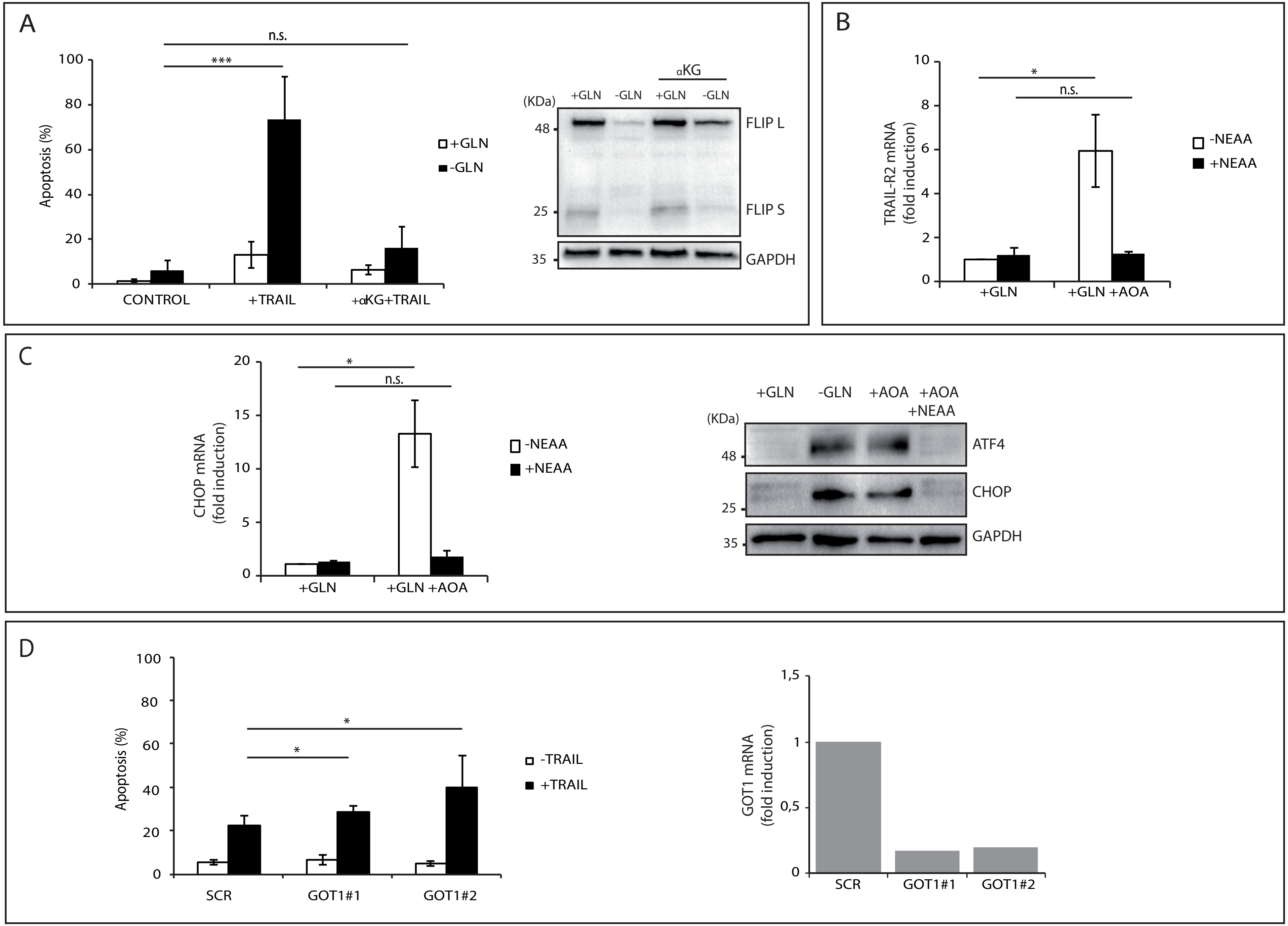
A
n.s.
***
100
80
60
40
20
0
Apoptosis (%)
+GLN
-GLN
CONTROL
+TRAIL
+αKG+TRAIL
αKG
(KDa)
+GLN
-GLN
+GLN
-GLN
48
25
35
FLIP L
FLIP S
GAPDH
B
10
8
6
4
2
0
*
n.s.
TRAIL-R2 mRNA
(fold induction)
-NEAA
+NEAA
+GLN
+GLN +AOA
C
20
15
10
5
0
*
n.s.
CHOP mRNA
(fold induction)
-NEAA
+NEAA
+GLN
+GLN +AOA
+GLN
-GLN
+AOA
+AOA
+NEAA
(KDa)
48
25
35
ATF4
CHOP
GAPDH
D
100
80
60
40
20
0
*
*
Apoptosis (%)
-TRAIL
+TRAIL
SCR
GOT1#1
GOT1#2
1,5
1
0,5
0
GOT1 mRNA
(fold induction)
SCR
GOT1#1
GOT1#2

Figure S3
